# Human hepatic 3D spheroids as a model for steatosis and insulin resistance

**DOI:** 10.1038/s41598-018-32722-6

**Published:** 2018-09-24

**Authors:** Mikael Kozyra, Inger Johansson, Åsa Nordling, Shahid Ullah, Volker M. Lauschke, Magnus Ingelman-Sundberg

**Affiliations:** 1 Section of Pharmacogenetics, Department of Physiology and Pharmacology, Karolinska Institutet, SE-17177 Stockholm, Sweden; 2 Division of Clinical Pharmacology, Department of Laboratory Medicine, Karolinska Institutet, and Karolinska University Hospital Laboratory Huddinge, SE-141 86 Stockholm, Sweden

## Abstract

Non-alcoholic fatty liver disease (NAFLD) has emerged as a public health concern as reflected in its widespread distribution in the general population. Yet, treatment options are scarce which is at least in part due to lack of reliable human *in vitro* disease models. Here, we report a human hepatic 3D spheroid system cultured under defined chemical conditions that has the potential to mimic steatotic conditions in a reversible manner, useful for identification of novel drug treatment conditions. Primary human hepatocytes (PHH) from different donors were cultured as spheroid microtissues in physiological *in vivo -*like culture conditions. Hepatic steatosis was induced over the course of three weeks in culture by supplementing the culture medium with pathophysiological concentrations of free fatty acids, carbohydrates and insulin. Effects of steatosis in the 3D system were evaluated on transcriptional, metabolomic and lipidomic levels. Free fatty acids on one hand as well as a combination of insulin and monosaccharides, promoted lipid accumulation in hepatocytes and increased expression of lipogenic genes, such as fatty acid synthase. This milieu also promoted development of insulin resistance within 2 weeks as manifested by an increase in gluconeogenic and insulin resistance markers, which are observed in type 2 diabetes mellitus and metabolic syndrome. Induced steatosis was reversible after withdrawal of lipogenic substrates and a further reduction in cellular fat content was observed following treatment with different antisteatotic compounds, such as metformin, glucagon, olaparib and antioxidants. Taken together, these results demonstrate that the 3D hepatic spheroids can serve as a valuable, HTS compatible model for the study of liver steatosis and facilitate translational discovery of novel drug targets.

## Introduction

Non-alcoholic fatty liver disease (NAFLD) is now one of the most prevalent liver diseases, affecting 20–50% of the general population^1,2^. The term encompasses a spectrum of liver diseases, including hepatic steatosis and non-alcoholic steatohepatitis (NASH), which can further progress to fibrosis, cirrhosis and hepatocellular carcinoma^1^. These conditions are hallmarked by the excessive accumulation of lipids (steatosis) which, by definition in steatosis, exceeds 5% of total liver weight^2,3^. Hepatic lipid accumulation is a key driver for disease initiation and gives rise to both morphological and functional perturbations of liver architecture and function^4^. Moreover, hepatic lipid perturbations are tightly connected to the metabolic syndrome, type 2 diabetes mellitus (T2DM) and dyslipidemia^5^. This interconnectivity is also reflected in the high prevalence of NAFLD among people who are overweight and/or suffer from T2DM^6^, and these conditions are therefore considered risk factors for NAFLD development^7^. Given the worldwide increase in obesity and consequently NAFLD prevalence^8,9^, this liver disease is predicted to be the leading cause of liver transplantation in the near future^10^.

The development of hepatic steatosis originates from an imbalance between lipid anabolism, catabolism and secretion. The human dietary intake of sugar, especially fructose, remains at high levels in the Western diet^11^, which has been shown to activate *de novo* lipogenesis via activation of the transcription factors ChREBP and SREBP1c^12^. The net result is intracellular lipid droplet formation^13^, which mediates formation of portal and lobular inflammation and infiltration of inflammatory cells and liver injury, jointly termed NASH^14^. Up to 20% of patients with steatosis have been found to have inflammation in the liver characterized by infiltration of macrophages, T lymphocytes, neutrophils, and dendritic cells^14^. Hepatic lipid accumulation also promotes development of insulin resistance^15^. Taken together, these events promote disease progression from simple steatosis to inflammation, fibrosis via activation of stellate cells and finally end-stage liver disease. However, the exact molecular events that underlie NAFLD disease progression are still being unraveled; lipid droplets and their biology have gained attention as they can be potential targets for therapeutics both in initial and advanced disease stages. Indeed, it has previously been shown that a decrease in steatosis in many cases is a prerequisite for resolution of steatohepatitis^16^, which highlights the importance of lipids as a crucial mediator in the pathogenesis of NAFLD.

Despite the widespread distribution and high prevalence of fatty liver disease, there is currently no therapy with regulatory approval. Even though diet and other lifestyle modifying approaches are corner-stones in current medical management, permanent weight-loss can only be expected in a fraction of patients due to low compliance^17^. Thus, there is an ongoing search for pharmaceutical intervention strategies that could target the key machinery of the disease. However, this activity has been partly hampered by the lack of translational and reliable disease models. The vast plethora of models for NAFLD, including animal models and *in vitro* cell models, capture mechanistically different aspects of the disease due to cellular phenotypes inappropriate for human liver *in vivo* and incorrect inducers for the disease, such as methionine-choline deficient diets^18,19^, which do not fully reflect human pathophysiology^20^.

Several *in vitro* models of fatty liver disease have been developed during the last decades. These include both scaffold-containing and scaffold-free systems. Examples of such systems are sandwich cultures, microfluidic devices and organ on a chip systems^21^. Drawbacks of many *in vitro* systems include the failure to support long-term experiments, lack of scalability, inappropriate cellular phenotype or unphysiological concentrations of lipogenic substrates and nutrients in standard cell media. Therefore, it is clear that there is a need for better *in vitro* models of the liver that could emulate the true pathophysiology of fatty liver disease.

Cultivation of primary human hepatocytes (PHH) in 3D spheroid configuration has recently been shown to closely mimic human liver function *in vitro*^22^. In this culture system, hepatocytes retain their cell-cell contacts, viability and mature hepatocyte phenotype as judged by proteome analysis^22^. Unlike conventional hepatocyte 2D cultures, the 3D culturing does not result in dedifferentiation and significant alterations of metabolic and signaling pathways, nor to a decrease in metabolic activity^22,23^. Furthermore, the 3D hepatic spheroids are viable, functional and stable over at least 35 days, as spheroids continue to secrete proteins, for example albumin^22,24^. Thus, hepatic 3D spheroids are an appealing tool for studies of the human liver in health and disease.

Here, we developed a model of human steatosis utilizing 3D hepatic spheroids in physiological and pathophysiological conditions. We demonstrate that 3D spheroids accumulate lipid droplets after exposure to excessive free fatty acid, carbohydrate and insulin levels. Moreover, the system captures many *in vivo* phenomena such as development of insulin resistance, reversibility of steatosis and successful treatment using different types of drugs. Taken together, this *in vitro* system could provide mechanistic insights into the pathogenesis of steatosis and insulin resistance and offer new perspectives on possible pharmaceutical targets.

## Methods

### Primary human hepatocyte 3D spheroid cultures

Cryopreserved primary human hepatocytes (obtained from Bioreclamation IVT, USA) were thawed according to the supplier’s instructions. The cells were subsequently seeded in ultra-low attachment (ULA) plates (Corning) at a density of 1,500 viable cells per well, as previously described^22^. Cells were seeded in Williams E medium (PAN-Biotech, Germany) supplemented with 5.5 mM glucose, 2 mM L-glutamine, 100 units/ml penicillin, 100 μg/ml streptomycin, 100 nM dexamethasone, 5.5 μg/ml transferrin, 6.7 ng/ml sodium selenite and 10% fetal bovine serum (FBS). The final concentration of insulin in the medium was 100 pM, unless otherwise stated. Donor and demographic information of the cell origins are shown in Table 1. After spheroid aggregation at day 5 after seeding, spheroids were further cultured in serum-free medium.

**Table 1:** Overview of commercially purchased primary human hepatocytes used in the experiments.

Donor	A	B	C	D
Sex	Male	Male	Female	Female
Age	22	58	48	30
Pathology	Carcinoid tumor of appendix	Cerebrovascular accident	Trauma	Trauma
Origin	Caucasian	Caucasian	Polynesian	Hispanic
BMI	22.6	25.7	21.2	30.8
Neutral lipids in healthy conditions*	1	6	1	1.5
Fold-increase in neutral lipids**	15	3	45	8

*Relative neutral lipids at day 14 in the healthy medium (0 µM FFA, 5.5 mM glucose, 0.1 nM insulin), normalized to donor A.

**Steatosis induction (14 days) with 320 µM FFA, 5.5 mM glucose, 0.1 nM insulin compared to control cultured in healthy medium with 0 µM FFA, 5.5 mM glucose, 0.1 nM insulin.

### Induction of steatosis

Exposure and treatment with lipogenic substrates started at day 7 after seeding of PHH when the spheroids had been formed, which hereafter is regarded to as day 0. Spheroids were exposed to either physiological medium (100 pM insulin, 5.5 mM glucose) or lipogenic media containing higher levels of insulin supplemented with free fatty acids and monosaccharides (glucose and fructose), see Results section. A combination (1:1 ratio) of the saturated palmitic acid (Sigma-Aldrich) and unsaturated oleic acid (Sigma-Aldrich) was used to mimic human plasma concentrations of free fatty acids. In order to facilitate free fatty acid uptake to the hepatic spheroids, the free fatty acids were bound to 10% bovine serum albumin (Sigma-Aldrich) at a molar ratio of 1:5 for 2 hours at 40 °C. Spheroids were exposed every 48–72 hours with the indicated substrates.

### Cell viability assay

The CellTiter Glo Luminescent Cell Viability Assay kit (Promega, Sweden) was utilized to measure ATP content and thereby cell viability according to manufacturer’s instructions. Briefly, 25 µl of the reagent was added to individual spheroids. After disruption of spheroids by pipetting, the plate was incubated at 37 °C in 5% CO_2_ for 20 minutes with subsequent luminescent signal measurement using MicroBeta LumiJET 2460 Microplate Counter (Perkin Elmer, USA).

### Microscopy of lipid accumulation

The NileRed lipid stain (Sigma-Aldrich) was used for fluorescence microscopy. Spheroids were fixed in 4% formaldehyde solution (Sigma-Aldrich) at room temperature for one hour and thereafter washed three times with PBS. Next, spheroids were incubated in 2 µM NileRed stain in PBS together with 1 µg/ml Hoechst 33342 (Thermo Fisher Scientific, USA) O/N at room temperature. Before imaging, spheroids were again washed three times with PBS. All fluorescent images were acquired using an LSM710 confocal microscope (Zeiss, Germany) and images were processed with ZEN lite 2012 analysis software (Zeiss, Germany). The intensity of neutral lipid staining was quantified and normalized for number of nuclei per spheroid using CellProfiler software.

### Lipid assay and lipid quantification

The AdipoRed Adipogenesis Assay Reagent (Lonza, Switzerland) specifically partitions into fat droplets. Here it was used to measure lipid accumulation according to manufacturer’s instructions. Briefly, AdipoRed Assay Reagent was added to single spheroids. Spheroids were disrupted by vigorous pipetting and fluorescence was thereafter measured (ex: 485, em: 572) in a whole-plate fluorometer (Spectra Max Gemini, Göteborgs Termometerfabrik, Sweden). The AdipoRed Assay Reagent was verified in relation to confocal picture triglyceride quantification with CellProfiler Software. No significant difference between confocal imaging quantification and the AdipoRed Assay Reagent could be observed (Supplemental Fig. 1).

### Lipidomic analysis

Freshly isolated PHH were compared to both physiological and pathophysiological conditions at 14 days after spheroid aggregation. In total, spheroids corresponding to 1,008,000 hepatocytes were harvested per condition. Spheroids were washed twice in PBS, transferred to Pyrex tubes (VWR) and were thereafter subject to Lipidomic Mass Spectrometry as previously described^25^.

### Immunohistochemistry

Spheroids were fixed in 4% paraformaldehyde at 4 °C O/N. Immunohistochemistry was performed on cryosections (8 μm) for CYP3A4 (PAP011, 1:5,000, Cypex Limited, United Kingdom). The donkey anti-rabbit Alexa Fluor 488 was used as secondary antibody. Slides were mounted with ProLong Gold Antifade Mountant with DAPI and fluorescence was assessed by confocal microscopy (Zeiss LSM 710).

### Western blot analysis

In order to isolate protein, spheroids were harvested with RIPA Lysis and Extraction buffer (Thermo Fisher Scientific) supplemented with cOmplete protease and PhosSTOP inhibitors (Roche). Aliquots of the protein homogenate were subjected to SDS-PAGE and Western blot analysis according to Karlgren *et al*.^26^ with minor modifications. AmershamProtran membrane (GE Healthcare Life Sciences, Little Chalfont, Buckinghamshire, UK) was used and visualization was performed using the Super Signal West Femto Chemiluminescent Substrate (Thermo Fisher Scientific Inc., Waltham, MA USA). Primary antibodies for GSK3β (#9832 CellSignal) and Phospho-GSK-3β (#9336 CellSignal) were used.

### Drug treatments

Pre-steatotic or post-steatotic spheroids were subject to drug treatment. Thus, both preventive effects as well as reversibility of steatotic spheroids were assessed for the indicated compounds. Steatosis was thereafter reversed with or without the indicated drugs dissolved in DMSO, except metformin which was dissolved in PBS. The indicated drugs were obtained from Sigma-Aldrich unless otherwise stated. The maximum final concentration of DMSO never exceeded 0.4%.

### RNA isolation and cDNA synthesis

RNA isolation was performed using the standard protocol of ZR-Duet DNA/RNA Mini Prep Kit (Zymo Research, United States). The RNA-concentration was determined using NanoDrop-1000 (Thermo Fisher Scientific, Wilmington, DE, USA). The isolated RNA was next reverse-transcribed to cDNA with SuperScript III reverse transcriptase (Invitrogen, United States) using Gene Amp PCR System 9700 (United States).

### Gene expression profiling

The amplification reactions were carried out in an Applied Biosystems 7500 Fast Real Time PCR (Thermo Fisher Scientific) using TaqMan Universal or SYBR Green mix. TaqMan and SybrGreen primers are specified in Supplemental Table 1. The comparative C_T_-method was used to determine the amount of target, normalized to endogenous reference 18S or TBP and relative to a calibrator (2^−ΔΔCt^)^27^. A dissociation step was performed in samples with SybrGreen primers to test the specificity of the PCR reaction.

### Albumin-ELISA

Media from healthy as well as pathological culture conditions were collected at the indicated timepoints and were thereafter subject to ELISA in order to measure albumin secretion of hepatic spheroids. ELISA was performed on Nunc Maxi-Sorp plates (ThermoFisher) in 100-µL volumes according to the standard protocol for the human albumin-ELISA kit (Bethyl laboratories, USA). Absorbance was measured at 450 nm (Spectra Max Plus, Göteborgs Termometerfabrik, Sweden).

### Untargeted metabolomics using High resolution – Mass spectrometry

Samples aimed for untargeted metabolomics were prepared as follows: PHH were seeded in 3D spheroid cultures as described above. Supernatants from the medium from >60 spheroid replicates were harvested and snap frozen at the indicated time points (see Results). The extracellular metabolites were then quantified using untargeted mass spectrometric analyses on an Orbitrap HR-MS System as previously described^23^.

### Statistical analyses

Mean and SEM were used for descriptive purposes. Statistical analyses were carried out using GraphPad Prism version 5 (GraphPad Software Inc. La Jolla, CA, USA) and Microsoft Excel. Significance levels for fatty liver disease induction experiments were calculated using the Student t test based on CellProfiler Software lipid quantification relative to spheroid size. P-value < 0.05 was considered significant.

### Study/ethics approval

Primary human hepatocytes used in the present study were commercial and thus no ethical approval was required.

## Results

### Modelling of hepatic environment for induction of steatosis

Hepatic 3D spheroids are liver-like culture systems which permit long-term cultivation of hepatic cells. In the present study, the hepatic spheroids consisted primarily of PHH, but also of small but detectable amounts of non-parenchymal cells (NPC:s) such as Kupffer and Stellate cells, as previously demonstrated by CD68 and vimentin staining respectively^22^. Each spheroid had a size of around 200 µm after aggregation. In order to reflect liver physiology in healthy and pathological conditions, culture media reflecting these respective conditions were designed. Physiological, “healthy”, medium contained 5.5 mM glucose and 0.1 nM insulin, whereas pathologic media consisted of high concentration of free fatty acids, insulin and the monosaccharides glucose and fructose, in line with *in vivo* human NAFLD data^28^.

**Figure 1:**
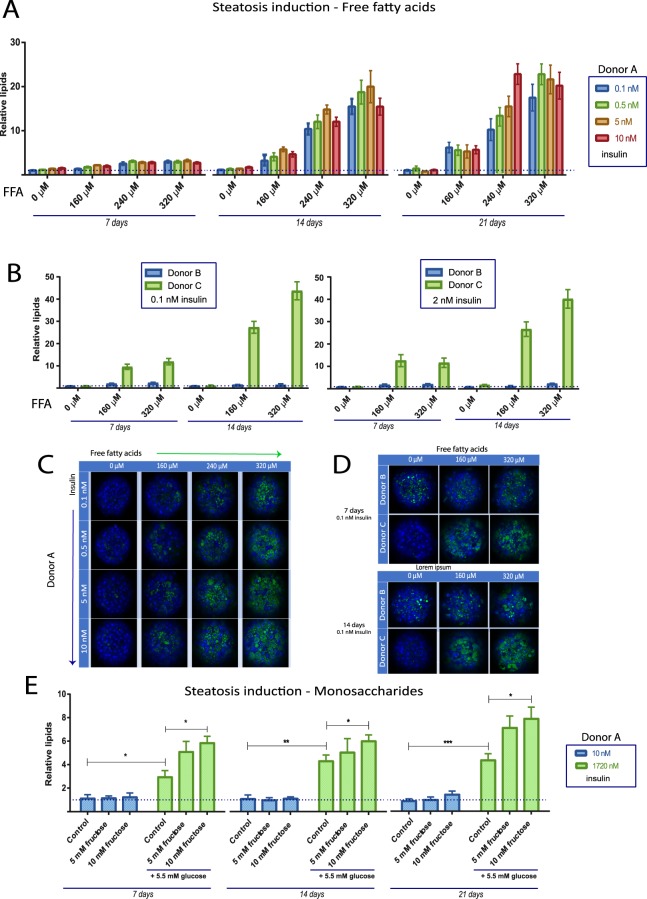
Induction of hepatic steatosis with free fatty acids, monosaccharides and insulin in three different donors. (**A**) Spheroids from donor A were treated with an equal mix of oleic and palmitic acid bound to albumin (final concentrations 160 µM, 240 µM and 320 µM respectively) as well as different concentrations of insulin (0.1 nM to 10 nM). Cell nuclei as well as lipid accumulation was visualized utilizing Hoechst 33342 and Nile Red staining. Neutral lipid content in each spheroid was quantified with CellProfiler Software and normalized to the size of each spheroid. (**B**) Induction of hepatic steatosis in donor B and C. The relative neutral lipid abundance in each donor is compared to the corresponding control of each donor, which was set to 1 for both donors. (**C**) Representative confocal images after induction of hepatic steatosis with free fatty acids and insulin after 14 days from donor A. (**D**) Representative picture of spheroids after induction of hepatic steatosis after 7 and 14 days in donor B and C. (**E**) Induction of hepatic steatosis with monosaccharides (glucose and fructose) and insulin. Abbreviations: FFA = Free fatty acids *p < 0.05, **p < 0.01, ***p < 0.001.

Initially we investigated the optimal conditions and timeframe for the induction of steatosis in the *in vitro* 3D liver system. Four donors where used to capture potential variability in induction of steatosis and variability in fat accumulation. PHH were seeded in physiologically healthy conditions and, following spheroid formation aggregation at day 7, they were exposed to oleic and palmitic acid (1:1) at various concentrations in the presence of different concentrations of insulin (0.1 nM to 10 nM). The pathologic environment with elevated free fatty acids (up to 320 µM total free fatty acids) promoted lipid accumulation and an increase in total fat content in donor A already after 7 days compared to control in healthy medium (Fig. 1A) but was highly accentuated after 14 and 21 days. The spheroids remained viable during this culture time as determined by measuring intracellular ATP (data not shown). The spheroids continued to secrete albumin in the pathologic conditions and no significant differences (mean ± SEM) in secreted albumin could be observed between healthy media (176 ± 17 ng/ml) and pathologic conditions (220 ± 38 ng/ml) after 14 days treatment. A clear FFA dose dependency of induction of steatosis could be observed (Fig. 1C). By contrast, the healthy culture conditions with physiological concentration of insulin and glucose did not cause lipid accumulation. The effect of insulin concentration on lipid accumulation was found to be of minor importance for steatosis induction in the presence of FFA. The distribution of the lipid droplets in the spheroids was found to be concentrated to particular areas in the hepatic spheroids following low free fatty acid exposure (160 µM), whereas higher concentrations of free fatty acids (320 µM) promoted steatosis induction in the majority of the cells in a spheroid (Fig. 1C).

Next, we evaluated induction of steatosis using three other donors (B, C and D). Donor C (Fig. 1B) and donor D (Supplemental Fig. 2) had very low total amount of lipids in healthy conditions, similar to donor A, and responded with excessive lipid accumulation following free fatty acid exposure. As with donor A, elevated insulin concentration was not found to significantly affect the FFA induced increase in triglyceride content. Donor C showed the highest susceptibility to steatosis development in terms of relative lipid accumulation compared to control. Hepatocytes from Donor B, the oldest donor (aged 58), were found to contain considerable amount of lipid droplets already as freshly isolated cells (>6-fold more fat compared to the youngest donor A, quantification not shown) (Fig. 1D). Consequently, only a very small extent of extra steatosis was here seen. The BMI score of the different donors was not found to correlate with the amount of lipids in healthy control conditions nor to the relative increase in lipids (Table 1). In summary, donor A, C and D were found to respond well to free fatty acid treatment and donor (A) was thereafter mainly used in subsequent experiments.

Having observed the induction of steatosis with free fatty acids, we investigated if the monosaccharides fructose and glucose could do the same. We found that these monosaccharides induce *de novo* lipogenesis and lipid accumulation in the presence of excessive insulin concentration (1,720 nM, standard Williams E medium) already after 7 days (>3-fold increase, P < 0.05) (Fig. 1E). As with free fatty acids, induction of steatosis with monosaccharides was time-dependent; 21 days in culture contributed to a higher lipid content compared to fewer days of steatosis induction with either glucose or fructose (Fig. 1E).

### Hepatic spheroids are sensitive to insulin and show signs of hepatic insulin resistance development

**Figure 2:**
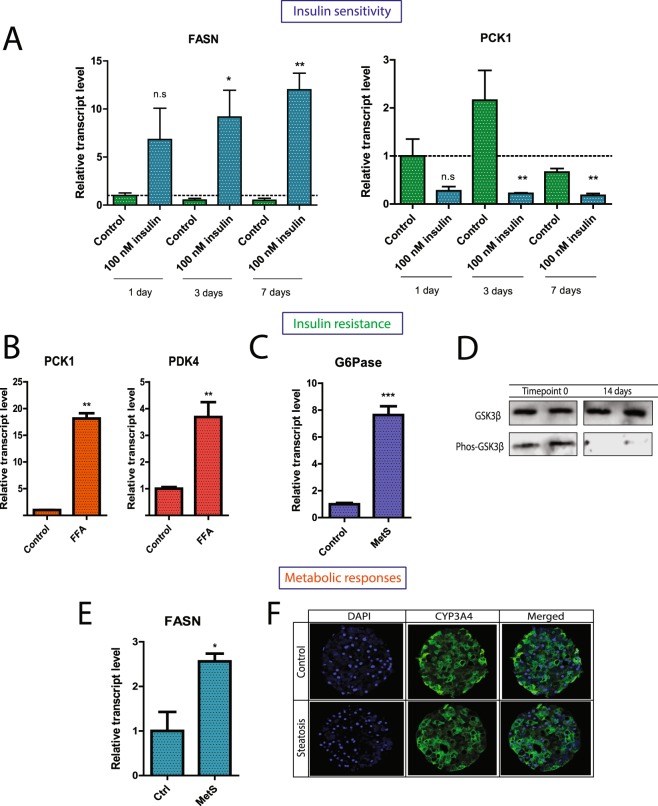
Spheroids are insulin sensitive, develop insulin resistance after induction of hepatic steatosis and express key lipogenic genes. (**A**) Spheroids from donor A were stimulated with 0.1 nM (control) or 100 nM insulin, respectively, every 24 hours and spheroid RNA was isolated after 1, 3 and 7 days respectively. FASN and PCK1 gene expression was assessed relative to control with qPCR normalized to the endogenous expression of the housekeeping RNA 18S. (**B**) Gene expression of PCK1 and PDK4 14 days after induction of steatosis. Steatosis was induced with 320 µM free fatty acids, and insulin concentration was held stable. (**C**) Gene expression of glucose-6-phosphatase (G6Pase) after 7 days in culture. MetS = ‘metabolic syndrome’: 11 mM glucose, 160 µM free fatty acids and 1,720 nM insulin. (**D**) Western Blot of steatotic spheroids revealed a clear reduction in phosphorylation of GSK3β after 14 days of treatment with 160 µM free fatty acids in addition to 11 mM glucose and 1,720 nM insulin. (**E**) FASN expression following induction of steatosis. (**F**) Staining of CYP3A4 in control and steatotic spheroids after 10 days revealed unaltered expression of CYP3A4 in the hepatic spheroids. Spheroids were treated with 320 µM free fatty acids in addition to 11 mM glucose and 1,720 nM insulin. Representative image from 3 replicates *p < 0.05, **p < 0.01, ***p < 0.001.

In addition to nutritional factors, hormones regulating intermediary metabolism can evoke activation of various transcription factors, gene products and enzymes involved in lipogenesis and lipolysis, for example insulin. To study the effects of insulin on the regulation of intermediary metabolism in the human liver, we exposed the hepatic spheroids to elevated concentration of insulin (100 nM) and assessed the responses at a transcriptional level. Upon insulin stimulation, the hepatic spheroids responded by at least a seven-fold increase (p < 0.05) in fatty acid synthase (FASN) transcription already after a few days in culture (Fig. 2A), suggesting that spheroids maintain their insulin signaling, are sensitive to insulin and induce pathways that are involved in lipogenesis. Besides stimulating lipogenesis, insulin decreases expression of gluconeogenic genes by activating Forkhead box protein O1 (FOXO1)^29^. The expression of phosphoenolpyruvate carboxykinase 1 (PCK1) mRNA, encoding the main checkpoint enzyme for the control of gluconeogenesis, was decreased by at least 70% (p < 0.01) compared to control after insulin stimulation (Fig. 2B), providing further evidence for the insulin sensitivity and physiological response of hepatic spheroids.

Following the observation of insulin sensitivity of hepatic spheroids, we next sought to evaluate whether the hepatic spheroids can mimic hepatic insulin resistance, an important hallmark of NAFLD^30^. To this end we investigated whether hepatic steatosis induction is associated with an increase in hepatic expression of genes associated with insulin resistance, for example an increase in hepatic gluconeogenesis and glycolysis. We found that PCK1 mRNA^31^ was induced in response to lipid accumulation after 14 days of steatosis induction (>15-fold increase, p < 0.01) (Fig. 2B). Moreover, pyruvate dehydrogenase lipoamide kinase isozyme 4 (PDK4) mRNA expression was increased, providing further signs of development of hepatic insulin resistance in the hepatic spheroids^32^ (Fig. 2B). Glucose-6-phosphatase mRNA, encoding an important enzyme for glycogenolysis and glucose production, was increased (>7-fold, p < 0.001) already after 7 days after steatosis induction with a combination of free fatty acids, glucose and insulin (Fig. 2C). Next, we assessed whether insulin resistance could be observed at the protein level. We thus investigated the response of protein kinase glycogen synthase kinase 3 (GSK3β) to insulin which is implicated in glycogen homeostasis in the liver^33^. After 14 days in the lipogenic environment, the hepatic spheroids in one donor examined developed insulin resistance as evident from a total reduction of GSK3β phosphorylation and thus a decrease in phos-GSK3β/GSK3β–ratio compared to control time point (Fig. 2D).

### Hepatic spheroids respond metabolically to steatosis induction and maintain their xenobiotic capacity

Induction of fatty liver disease is characterized by changes in expression of various genes, for example those involved in intermediary metabolism. In order to assess such changes in steatotic spheroids, we measured the expression of FASN mRNA, encoding a vital enzyme in lipogenesis (Fig. 2E). We found that a combination of free fatty acids, monosaccharides and insulin (‘metabolic syndrome’) together resulted in a >2-fold increase in FASN mRNA expression (p < 0.05).

The level of CYP3A4 protein expression was stable after induction of steatosis by free fatty acids indicating the retained differentiation state of the hepatocytes including capacity for drug metabolism by this clinically important cytochrome P450 (Fig. 2F).

### Lipidomic signatures in steatotic spheroids

Steatotic livers are characterized by altered lipidomic profile compared to healthy livers. In order to investigate the lipidomic signatures and the alterations of individual lipid species after induction of fatty liver disease, samples from steatotic and non-steatotic spheroids were subject to lipidomic analysis. In addition, freshly isolated cells before seeding of spheroids were subject to analysis to determine culture effects of steatotic spheroids on the lipidomic profile. Steatosis was induced by cell culture media which consisted of elevated levels of free fatty acids, monosaccharides and insulin to reflect the *in vivo* environment the liver is exposed to in patients with the metabolic syndrome.

**Figure 3:**
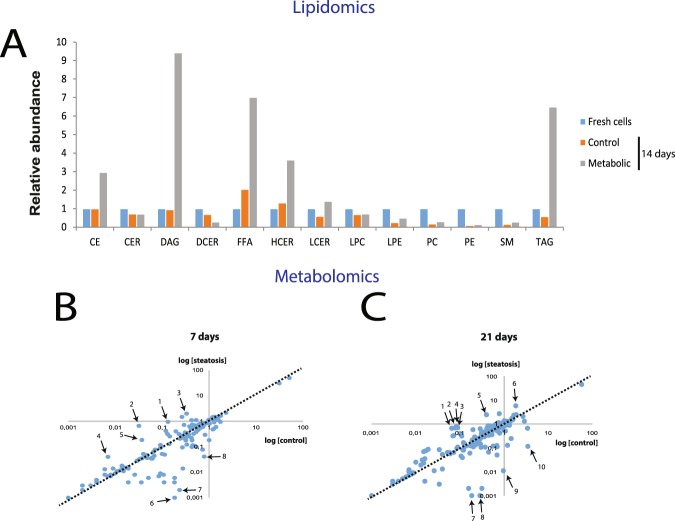
Lipidomic and metabolomic profiles of hepatic spheroids after induction of fatty liver disease. (**A**) Lipidomic analysis of fresh cells from donor A as well as spheroids exposed to either control (healthy) or pathological media (“metabolic syndrome”) for 14 days after spheroid formation. In total, spheroids corresponding to 1,008,000 cells were harvested for each sample. Control medium contained 0.1 nM insulin and 5.5 mM glucose, whereas “metabolic syndrome” conditions consisted of 10 nM insulin, 5.5 mM glucose, 10 mM fructose and 320 µM free fatty acids (1:1 oleic and palmitic acid). Abbreviations: CE = cholesterol esters, CER = ceramide, DAG = diglyceride, DCER = dihydroceramide, FFA = free fatty acids, HCER = hydroxyceramide, LCER = lactosylceramide, LPC = lysophosphatidylcholine, LPE = lysophosphosphatidylethanolamine, PC = phosphatidylcholine, PE = phosphatidylethanolamine, SM = sphingomyelin). (**B**) Metabolomic analysis of secreted extracellular metabolites at 7 days after spheroid formation from donor A. Steatosis was induced with elevated insulin (10 nM) and 320 µM FFA. In total 147 unique metabolites were identified. The relative abundance of the metabolites in the two respective conditions are presented as logarithmic values. The outliers are specified in Supplemental Table 2. (**C**) Metabolomic analysis at 21 days after spheroid formation from donor A. Outliers are specified in Supplemental Table 3.

In total, 13 lipid classes were analyzed, specified in Fig. 3A, and revealed many differences between control and metabolic syndrome samples. The majority of lipid classes were upregulated in metabolic syndrome–conditions compared to control; the three most upregulated being diglycerides, free fatty acids as well as triglycerides. The lipidomics also revealed that the relative abundance of lipid classes is stable in the healthy culture conditions compared to freshly isolated cells, including triglycerides and ceramides. Nevertheless, 4 different phospholipids were downregulated in the spheroids cultivated in the healthy medium compared to freshly isolated hepatocytes, indicating a direct culture effect on phospholipid composition of hepatic spheroids.

### Endogenous metabolic signatures after induction of hepatic steatosis

Currently, we lack reliable biomarkers of fatty liver disease in a clinical setting^34^. In order to assess the metabolic profiles of steatotic spheroids and pinpoint potential biomarker candidates, the endogenous metabolites produced in the hepatic spheroid system following fatty liver disease induction were measured. The media from the spheroids were harvested and measured for metabolites after 7 and 21 days after aggregation respectively. The metabolomic analysis revealed that hepatic spheroids are more or less metabolically stable over the indicated time period (Fig. 3B). However, some metabolites were found to differ between control and steatotic samples at 7 (Fig. 3B) and 21 days (Fig. 3C) respectively as shown in Supplemental Tables 2 and 3. Some of the putative most upregulated metabolites after 21 days in steatotic culture conditions were identified based on their MW as 3-ethoxy-N-(tetrahydro-2-furanylmethyl)-propanamide (>60-fold increase), 7-chloro-2-thioxo-2,3-dihydro-4H-pyrido[1,2-a][1,3,5]triazin-4-one (>60-fold increase) and (6E)-8-oxogeranial (>60-fold increase). Further investigations will reveal whether these could constitute biomarkers for steatosis *in vivo* in man.

### Hepatic steatosis is reversible in hepatic spheroids and the system responds to drug treatment

**Figure 4:**
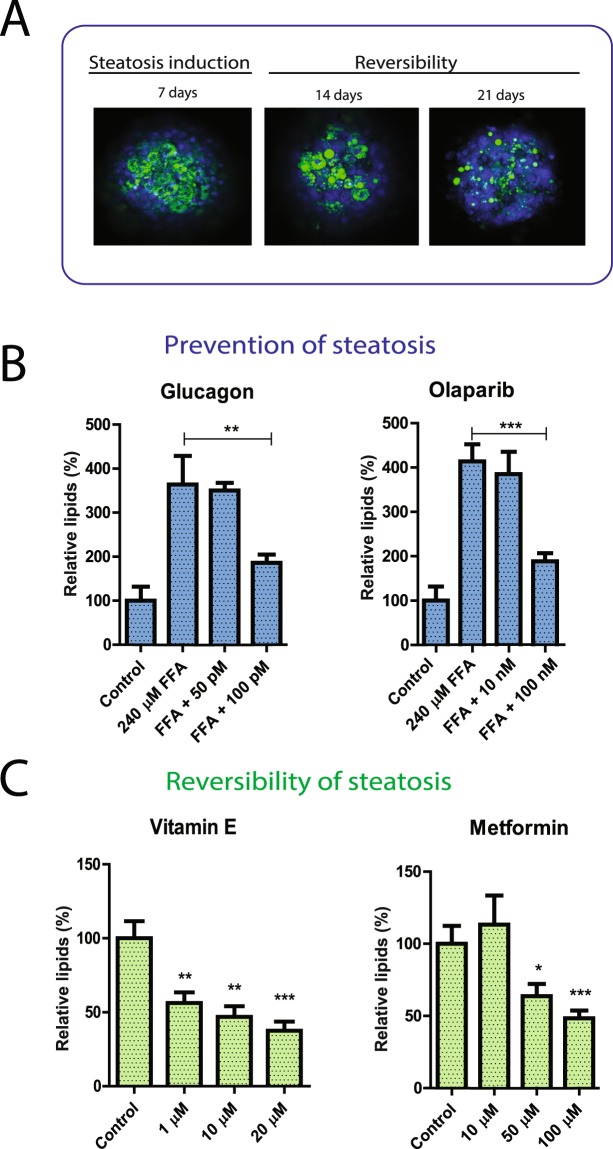
Steatosis can be reversed in hepatic spheroids and successfully treated with various types of drugs. (**A**) Reversal of induced hepatic steatosis with healthy medium. Spheroids from donor A were made steatotic by treatment with elevated concentration of free fatty acids (320 µM) for seven days and the spheroids were thereafter cultivated in the healthy medium (0.1 nM insulin, 5.5 mM glucose) in the absence of free fatty acids for 14 days. Cell nuclei as well as triglyceride accumulation was visualized utilizing Hoechst 33342 and Nile Red staining after 7, 14 and 21 days. The pictures are representative images of two independent experiments. (**B**) Prevention of steatosis by co-treatment with glucagon (50 and 100 pM) and olaparib (10 and 100 nM). Spheroids from donor A were treated with glucagon and olaparib for 10 days in the presence of 240 µM free fatty acids. Lipid levels were quantified using AdipoRed biochemical quantification assay in two independent experiments. (**C**) Reversibility of steatosis. Hepatic spheroids from donor A were made steatotic by treatment with 320 µM free fatty acids during 7 days. The spheroids were thereafter cultivated in medium without free fatty acids with or without vitamin E and metformin. Lipid levels were quantified using both AdipoRed biochemical quantification assay and confocal microscopy in two independent experiments. The effect of drug treatment is related to the corresponding control without drugs of steatosis stimulating factors. Data are results from the AdipoRed biochemical quantification assay *p < 0.05, **p < 0.01, ***p < 0.001.

Having observed induction of hepatic steatosis and accumulation of triglycerides, we next investigated whether the steatosis could be reversed in the hepatic spheroids. Steatosis was first induced during one week with 320 µM free fatty acids and thereafter the medium was deprived of free fatty acids for the rest of the culture time (Fig. 4A). We found that the lipid content in the spheroids declined slowly after medium change and after 14 days cultivation in the control medium, the lipid content approached the control levels.

Lastly, in order to validate whether the 3D hepatic spheroid system would constitute a suitable drug-screening platform, we explored whether the lipid content could be reduced by antisteatotic compounds that have previously shown such properties *in vivo*, including vitamin E^35^ metformin^36^. Moreover, we also investigated also whether glucagon increased lipolysis in the liver^37^ and in addition whether inhibition of poly ADP-ribosylation with the new anti-cancer drug olaparib, a PARP inhibitor, could prevent steatosis^38^. The compounds were assessed for their preventive effect on lipid accumulation as well as for treatment of steatosis in the case for vitamin E and metformin. Clinically relevant concentrations of the drugs were used to mimic therapeutic doses that are seen *in vivo*. We found that glucagon and olaparib partially prevented the development of fatty liver disease following an overload of fatty free acids in the medium (Fig. 4B). Neither metformin nor vitamin E at physiologically relevant concentrations prevented steatosis induction (data not shown). By contrast, both compounds significantly decreased the induced steatosis by at least 40% (p < 0.05) compared to untreated control following one-week induction of steatosis and subsequent one-week treatment with the indicated compounds (Fig. 4C).

## Discussion

This study aimed at developing a model of steatosis and insulin resistance utilizing hepatic 3D spheroids, consisting of only 1,500 cells in a chemically defined medium, primarily human PHH but also detectable amounts of non-parenchymal cells. Physiological and pathophysiological media were initially designed in order to mimic both healthy and pathological hepatic conditions. We demonstrate that hepatic spheroids from different donors of various age and original phenotype are capable of mimicking the pathological condition of hepatic steatosis, excess di- and triglyceride accumulation in the liver, following treatment with various nutrients and insulin. Importantly, this system captures many *in vivo* phenomena such as the development of insulin resistance and, importantly, reversibility of steatosis which indicates the spheroids suitable as an *in vitro* model to study fatty liver disease.

NAFLD is an emerging health issue globally, affecting a large proportion of the population in many countries^39^. Thus, several *in vivo* as well as *in vitro* models for liver disease studies and drug discovery have been developed during the last decades^21,40^. Human *in vitro* models are an important complement to animal models due the species differences in for example disease pathogenesis and drug response. Modelling of disease *in vitro* could thus offer many advantages in case of diseases being species-specific. Moreover, *in vitro* experiments offer the advantage of conducting high-throughput experiments encompassing a multitude of different compounds and conditions of relevance for the disease and the treatment. However, many challenges with *in vitro* models are at hand, which must be capable of interrogating and emulating many disease phenomena at the same time. For example, the models must exhibit *in vivo*-like properties, maintain their original *in vivo* phenotype and tissue-specific gene expression. Moreover, *in vitro* models should also elicit metabolic responses, for example retain their insulin sensitivity but also show signs of insulin resistance in pathophysiological conditions. Technological advancements have allowed the development of new models that aim to address these problems, with various success^21^. These technologies include different microfluidic chips, micropatterning, layered co-cultures or organ bioprinting^41–44^. Some of these models incorporate micro-circulation as well as biomechanical support which gives rise to more physiological micro-environments. The longevity of these systems vary from a couple of days in culture to multiple weeks^21^. Some of these systems try to mimic the hepatic microenvironment, but most of the systems lack validation of the hepatic phenotype.

Cultivation of PHH in 3D configuration is an appealing tool since the phenotype of the hepatocytes has been shown to be maintained^22^. Moreover, this hepatic spheroid model has successfully been shown to be usable for studies of bile accumulation and cholestatic disease^45^. As compared to the 3D spheroid system, other similar liver platforms use a much higher number of cells in the experimental setup, which limit scalability of the systems^41,42,46^. In our system, we have introduced a chemically defined serum free medium, which allows a firm evaluation for different endocrine and nutrient factors that affect liver disease. Thus, in the present study, the spheroids were exposed to serum-free culture conditions that allows to precisely tune the system, e.g. by supplementing the media with various substances, including free fatty acids, sugars, hormones as well as other molecular compounds. The formation of steatosis in the *in vitro* system requires control of the insulin, FFA and monosaccharide concentrations at relevant physiological levels impossible to achieve in many common commercial cell media which can alter drug metabolism pathways and insulin sensitivity^47,48^.

The induction of hepatic steatosis was achieved using both free fatty acids as well as a combination with monosaccharides and insulin. In order to mimic elevated plasma concentration of free fatty acids, which *in vivo* are mainly derived from the diet and adipose tissue^49^, we treated the spheroids with a combination of palmitic and oleic acids. These long-chain free fatty acids have previously been shown to accumulate in human hepatic steatosis^50^, which have also been used in many other systems^41,42^. We also treated the spheroids with the monosaccharides glucose and fructose, which in combination with insulin gave rise to lipid accumulation *in vivo*. Fructose has recently gained much attention since it is regarded as an important driver for lipogenesis and ectopic lipid accumulation in hepatocytes^51^, especially in the presence of higher levels of insulin. For this purpose, the monosaccharide fructose was used together with supraphysiological concentrations of insulin to induce steatosis. Following treatment with free fatty acids, monosaccharides and insulin, the spheroids responded by phenotypic alterations observed in patients suffering from NAFLD; lipid accumulation, alterations in hepatic expression of lipogenic genes (FASN) as well as development of hepatic insulin resistance. Indeed, the spheroids remained insulin sensitive in healthy cell media as evident from the physiological responses of FASN and PCK1 following insulin stimulation. By contrast, the hepatic spheroids showed signs of development of insulin resistance following induction of fatty liver disease, both at transcriptional level as well as protein level as evident from the increased level of PDK4, PCK1 and G6Pase as well as total reduction in the phosphorylation of GSK3β. It is indeed important to take the insulin resistance into consideration when characterizing different hepatic 3D models which is not always done. Insulin resistance is believed to be a central mechanism in NAFLD that leads to endoplasmic reticulum stress, lipid-derived toxicity as well as disturbed autophagy^39^. Together, these events cause hepatocyte injury, which is an important hallmark in the more severe stages of NAFLD^39^.

Interestingly, the four different donors responded differently to steatosis induction. Thus, it is evident that there is a large inter-individual variation in the capability for steatosis development in the spheroids. The relative extent of steatosis induction compared to the respective controls in each donor was found to differ, suggesting an intrinsic donor susceptibility. This could for example be due to different genotypes influencing the susceptibility to steatosis development^52^. Other explanations include age and underlying hepatic disease in the different donors. Furthermore, the results indicate that cells from donors carrying hepatocytes which were steatotic from the beginning, responded with less relative lipid accumulation after lipid promoting treatment.

Hepatic lipids are an important mediator of NAFLD induction and progression^39^, and the disease is associated with numerous changes in hepatic lipid composition^50^. Therefore, we analyzed the lipidome of hepatic spheroids following induction of steatosis. We demonstrated that hepatic spheroids accumulate triglycerides and diglycerides as evident from the lipidomic analysis. Interestingly, diglycerides was one of the most abundant lipid classes in steatotic conditions, which is the lipid class believed to cause insulin resistance^53^. Previous clinical studies have shown that, in line with our results, triglycerides and diglycerides are the main lipid classes, which are increased in fatty liver disease^50^.

As of today, we lack approved pharmaceutical treatment of NASH and NAFLD. There are nevertheless numerous clinical trials ongoing with drugs that target specific pathomechanisms in NAFLD^2,39^. An important feature of *in vitro* models of hepatic disease is that they should be able to predict drug response and serve as a translational tool for prediction of *in vivo* responses in human. Therefore, we investigated whether spheroids could be used as a screening tool for novel treatment of hepatic disease. We found that the induced steatosis is indeed reversible in hepatic spheroids and the levels of lipids can be diminished by drugs that have previously been shown to have lipid lowering effect either *in vivo* or *in vitro*^35–38^. Vitamin E and metformin accelerated reversal when spheroids were exposed to healthy media without free fatty acids, suggesting that these drugs have a synergistic effect together with a healthier plasma environment.

In summary, the present study indicates that the 3D liver spheroid system elicits many *in vivo* phenomena such as insulin resistance and, importantly, reversibility of steatosis, which makes the system suitable for the study of lipid droplets in biology and disease. The promising *in vivo* like features of the *in vitro* 3D liver system presented here in combination with the HTS performance and chemically defined medium would enable deeper studies into the molecular mechanisms and pathogenesis of fatty liver disease. Moreover, the system has the potential to facilitate development of new intervention strategies for hepatic liver disease. In conclusion, this study indicates that hepatic spheroids can be a useful preclinical model for fatty liver disease with translational implications.

## Electronic supplementary material

Supplementary information
