# Supplementary material for: Human hepatic 3D spheroids as a model for steatosis and insulin resistance
Source: Sci Rep. 2018 Sep 24;8:14297. doi: 10.1038/s41598-018-32722-6 (PMC6155201; doi:10.1038/s41598-018-32722-6)
Supplement: Supplementary file 1 — Supplementary information [file 41598_2018_32722_MOESM1_ESM.docx]

Supplementary Information Kozyra et al SREP-18-23708

Title: Human hepatic 3D spheroids as a model for steatosis and insulin resistance

Authors: Mikael Kozyra^1^, Inger Johansson^1^, Åsa Nordling^1^, Shahid Ullah^2^, Volker M. Lauschke^1^, Magnus Ingelman-Sundberg^1^

^1/^ Section of Pharmacogenetics, Department of Physiology and Pharmacology, Karolinska Institutet, SE-17177 Stockholm, Sweden

^2/^ Division of Clinical Pharmacology, Department of Laboratory Medicine, Karolinska Institutet, and Karolinska University Hospital Laboratory Huddinge SE-141 86 Stockholm, Sweden.

# .

**Supplementary Table 1:** Overview of TaqMan and SybrGreen primers used in the present study

| Gene | Taqman/SybrGreen sequence |
| --- | --- |
| FASN | Taqman (Hs01005622_m1) |
| PCK1  Forward primer:  Reverse primer: | SybrGreen  ACTCGAGGTTCTGCACCCCT  AGGCAGCATCAATGATGGG |
| PDK4 | Taqman (Hs01037712_m1) |
| G6Pase | Taqman (Hs02560787_s1) |

**Supplementary Table 2**: List of secreted metabolites in hepatocyte media different between healthy and lipid promoting media conditions as revealed by outliers from the metabolomic analysis after 7 days. Only metabolites that differed the most between the two culture conditions are in the list (see also Figure 3B for corresponding metabolite number).

| Metabolite | Number | Ratio [steatosis]/[control] |
| --- | --- | --- |
| N-[4-(4-Bromophenyl)-5-methyl-1,3-thiazol-2-yl]-2-pyridinamine | 1 | Increased |
| (3E)-4-(4,5-Dibromo-3-thienyl)-3-buten-2-one | 2 | Increased |
| 4-Chlorothieno[3,2-d]pyrimidine-6-carboxamide | 3 | Increased |
| N''-(1-Cyclopropylethylidene)carbonohydrazonic diamide | 4 | Increased |
| 2-(1H-Benzimidazol-2-ylsulfanyl)-N-(4-methyl-1,3-benzothiazol-2-yl)acetamide | 5 | Increased |
| 4-({3-[(4-Chloro-3-nitrobenzoyl)amino]phenyl}amino)-8-hydroxy-N-(4-methoxybenzyl)-3-quinolinecarboxamide | 6 | Decreased |
| Ethyl 5-carbamoyl-2-[(ethoxycarbonyl)amino]-4-methyl-3-thiophenecarboxylate | 7 | Deceased |
| 10-Hydroxy-10H-phenoxaphosphinine-2,8-dicarboxylic acid 10-oxide | 8 | Decreased |

**Supplementary Table 3:** List of secreted metabolites in hepatocyte media different between healthy and lipid promoting media conditions as revealed by outliers from the metabolomic analysis after 21 days. Only metabolites that differed the most between the two culture conditions are in the list (see also Figure 3C for corresponding metabolite number).

| Metabolite | Number | Ratio [steatosis]/[control] |
| --- | --- | --- |
| 3-Ethoxy-N-(tetrahydro-2-furanylmethyl)propanamide | 1 | Increased |
| 7-Chloro-2-thioxo-2,3-dihydro-4H-pyrido[1,2-a][1,3,5]triazin-4-one | 2 | Increased |
| (6E)-8-oxogeranial | 3 | Increased |
| 3,5-Dibromo-1-[2-(4-chlorophenyl)-2-oxoethyl]pyridinium bromide | 4 | Increased |
| 7-Chloro-2-thioxo-2,3-dihydro-4H-pyrido[1,2-a][1,3,5]triazin-4-one | 5 | Increased |
| 5,5,5-Trichloro-N-(2-methylphenyl)-1-pentanesulfonamide | 6 | Increased |
| 2-Oxo-2-(2-thienyl)ethyl 5-nitro-2-furoate | 7 | Decreased |
| Methyl 2-(3-chloro-2,5-dioxo-2,5-dihydro-1H-pyrrol-1-yl)-3-thiophenecarboxylate | 8 | Decreased |
| Methyl 1-(1H-imidazol-2-yl)-1H-1,2,3-triazole-4-carboxylate | 9 | Decreased |
| 2-(3-Pyridinyl)pyrrolidinium perchlorate | 10 | Decreased |


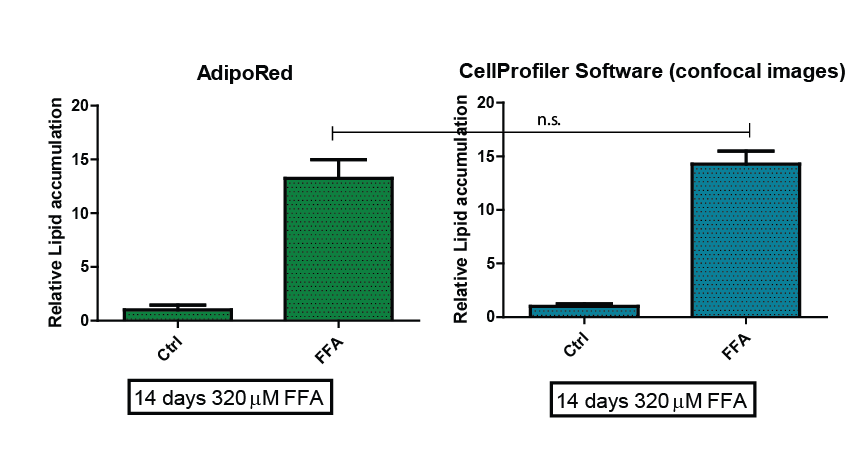
**Supplemental Figure 1.**

**A**

**B**

**FFA**

**Control**


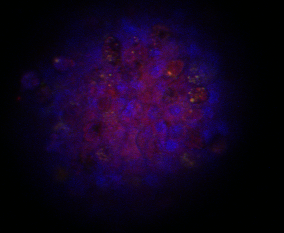

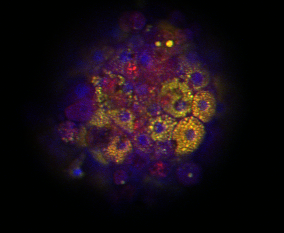


**Comparison between CellProfiler Software confocal image lipid quantification and the biochemical assay AdipoRed**. A) Hepatic spheroids were treated with 320 μM free fatty acids for 14 days and the relative induction of steatosis was quantified with the two methods. AdipoRed = 24 spheroids quantified. Confocal images = >7 spheroids quantified. B) Representative confocal images of the quantified spheroids. Abbreviations: FFA = free fatty acids

**Supplemental Figure 2.**

**
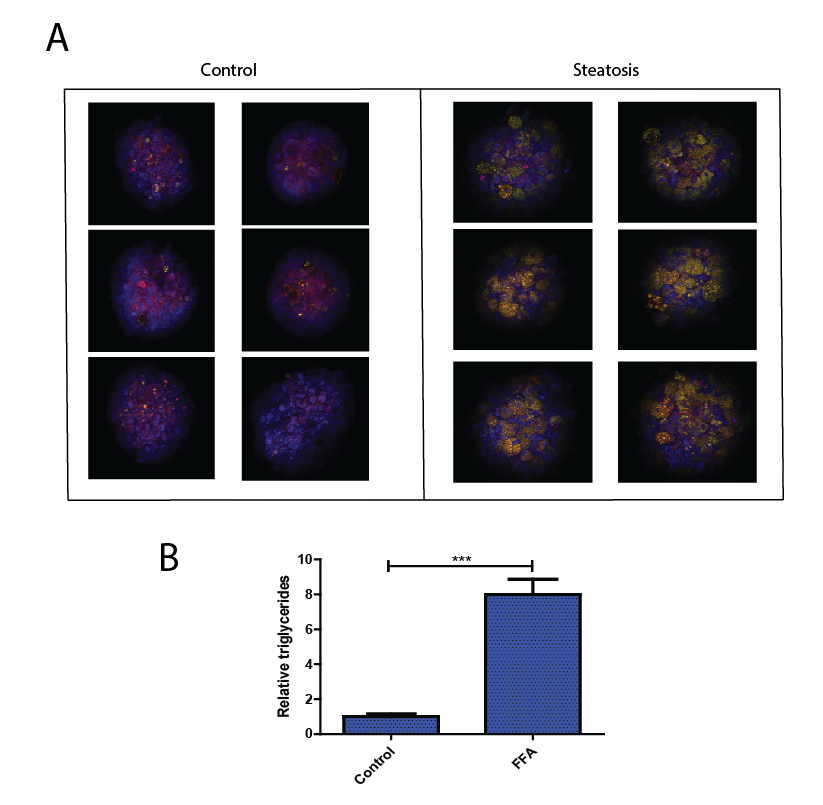
**

**Induction of hepatic steatosis in donor D A)** Spheroids from donor D were treated for two weeks with 320 μM FFA (1:1 palmitic and oleic acid). **B)** Quantification based on confocal images (CellProfiler Software) reveals an 8-fold increase in the relative triglyceride levels (p<0.001) in FFA-treated spheroids compared to control. Quantification based on > 6 images


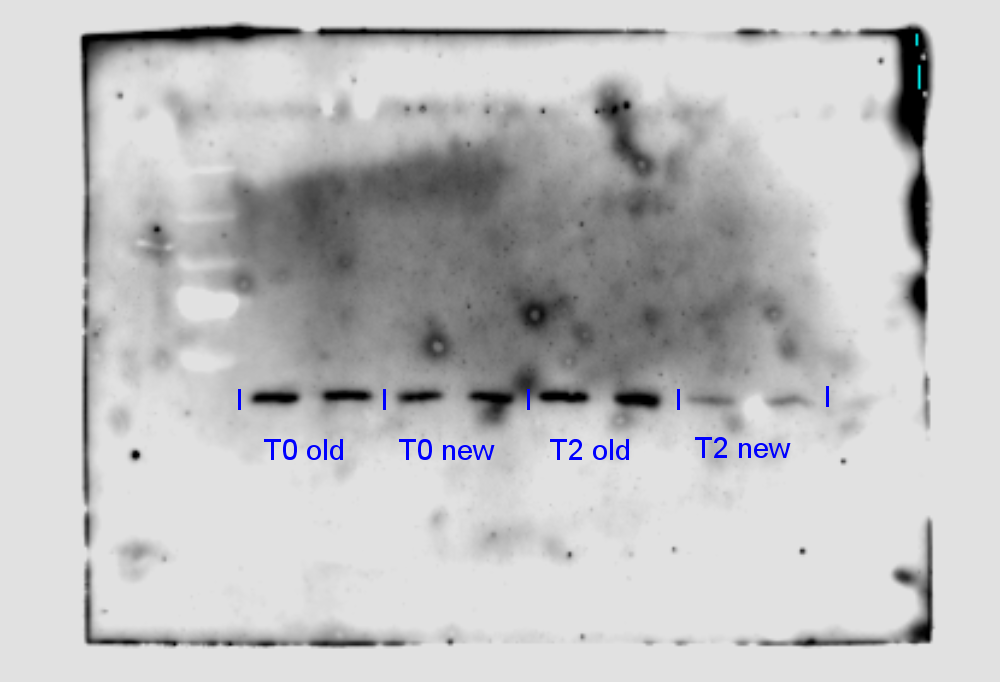


GSK3β


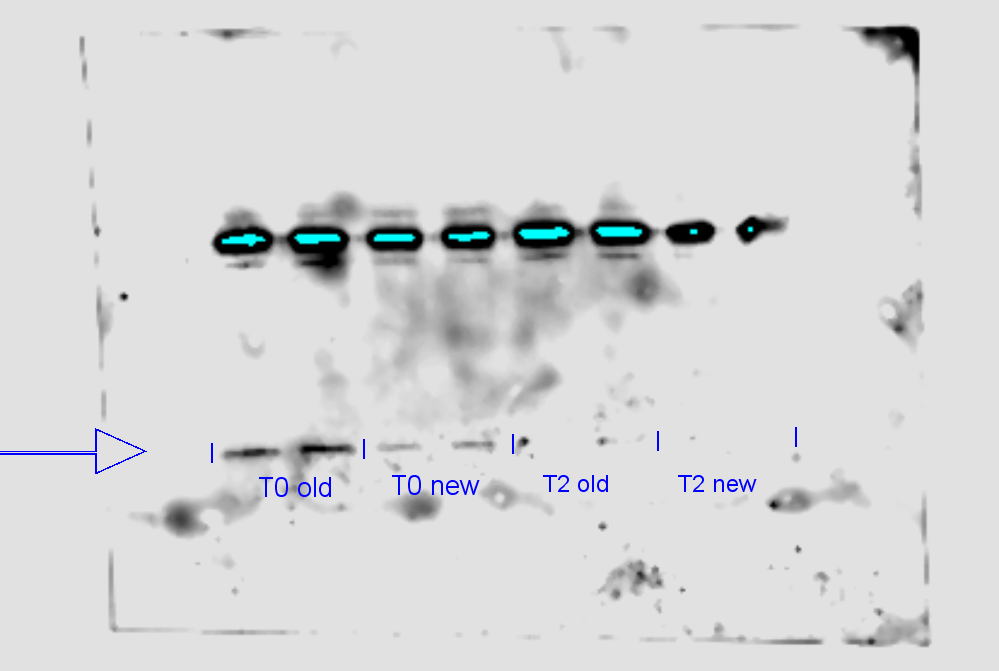


P-GSK3β

**Western Blot (uncropped gel from Figure 2D)** Abbreviations: T0 = Timepoint 0, T2 = 14 days, Old and new represents two different experiments
